# Mechanisms of Endometrioma-Mediated Ovarian Damage: Myths and Facts

**DOI:** 10.3390/jcm14072147

**Published:** 2025-03-21

**Authors:** Pınar Özcan, Bulut Varlı, Ertan Sarıdoğan, Engin Oral, Muhammed Mabrouk, Taner Usta, Alin Stefan Constantin

**Affiliations:** 1 Department of Obstetrics and Gynecology, Uskudar University School of Medicine, 34768 Istanbul, Türkiye; 2 Department of Obstetrics and Gynecology, Ankara University School of Medicine, 06620 Ankara, Türkiye; bvarli@ankara.edu.tr; 3 Women’s Health Division, University College London Hospital, London WC1E 6BT, UK; ertan.saridogan@nhs.net (E.S.); mohamed.mabrouk2@gmail.com (M.M.); 4 Department of Obstetrics and Gynecology, Biruni University School of Medicine, 34015 Istanbul, Türkiye; drenginoral@gmail.com; 5 Cleveland Clinic London Hospital, London SW1X 7HY, UK; 6 Department of Obstetrics and Gynecology, Acibadem University, Altunizade Hospital, 34662 Istanbul, Türkiye; drtanerusta@gmail.com; 7 Department of Obstetrics and Gynecology, Saarland University Hospital, 66421 Hamburg, Germany; constantinalin@gmail.com; 8 Department of Obstetrics and Gynecology, Carol Davila’ University of Medicine and Pharmacy, 050474 Bucharest, Romania

**Keywords:** endometrioma, ovarian reserve, in vitro fertilization, infertility, endometriosis

## Abstract

Ovarian endometriomas (OEMs), cystic formations within the ovaries, are a significant manifestation of endometriosis and present in 20–40% of affected women. Despite extensive research, the pathogenesis of endometriosis remains unclear, with retrograde menstruation, coelomic metaplasia, and lymphatic dissemination being proposed mechanisms. OEMs negatively impact ovarian function by reducing the ovarian reserve, disrupting folliculogenesis, and altering the ovarian microenvironment through oxidative stress, inflammation, and fibrosis. Elevated reactive oxygen species (ROS) accelerate follicular atresia, and extracellular matrix remodeling contributes to ovarian damage, while immune dysregulation and cytokine imbalances further exacerbate the condition. The presence of OEMs does not significantly affect live birth rates in in vitro fertilization (IVF) treatments, despite potential reductions in the quality and quantity of oocytes. However, their surgical excision compromises the ovarian reserve. This review highlights the complex mechanisms by which OEMs impair ovarian function and emphasizes the need for further research to develop strategies that mitigate these effects, ultimately improving reproductive outcomes for women with endometriomas.

## 1. Introduction

Endometriosis is a complex and chronic inflammatory condition characterized by the presence of endometrial-like tissue outside the uterus. This ectopic tissue primarily affects the ovaries, peritoneum, and other pelvic structures, significantly impacting the health, fertility, and quality of life of those affected. Within the broader context of endometriosis, an ovarian endometriotic cyst, or endometrioma, refers to cystic formations that develop within the ovaries due to the growth of ectopic endometrial tissue. Endometriomas represent a significant manifestation of endometriosis and are diagnosed in approximately 20% to 40% of women suffering from the condition [1,2]. There are several theories on the pathogenesis of endometriomas [3], including the following: invagination and subsequent collection of menstrual debris from endometriotic implants, which are located on the ovarian surface and adherent peritoneum [4]; colonization of functional ovarian cysts by endometriotic cells [5]; and coelomic metaplasia of the invaginated epithelial inclusions [6]. These lesions are frequently associated with pelvic pain and infertility, thereby making them a crucial focus of reproductive health research [7,8,9]. In this review article, we summarize the mechanisms of endometrioma-mediated ovarian damage based on the pathophysiology of endometrioma, including the changes in the local ovarian microenvironment.

## 2. Materials and Methods

A systematic search of electronic databases, including PubMed, Scopus, Web of Science, and Embase, was performed. Keywords and MeSH terms used included “ovarian endometrioma”, “ovarian reserve”, “anti-Müllerian hormone”, “antral follicle count”, “folliculogenesis”, “ovarian function”, and “fertility outcomes”. Only English-language publications released between January 2000 and December 2024 were included in the search. Studies were included if they investigated the effect of ovarian endometriomas on ovarian reserve markers, histological changes in ovarian tissue, or clinical fertility outcomes. Both observational studies (prospective and retrospective), interventional studies, and review articles were considered. Case reports and studies lacking ovarian function data were excluded. Two independent reviewers (P.Ö and B.V.) screened titles and abstracts, followed by full-text reviews to determine eligibility. Data extracted included the study design, sample size, patient characteristics, ovarian reserve markers, histopathological findings, and reproductive outcomes.

## 3. The Impact of Endometriomas on Ovarian Function

Endometriosis doubles the risk of infertility in women who are diagnosed with the condition before they start trying for a pregnancy [9]. This is likely to be due to a number of factors, including mechanical distortion, disturbances in ovum pick-up, and gamete and embryo transport. OEMs may be partly involved in these mechanisms. However, the influence of OEMs on ovarian function extends well beyond the mere presence of cysts. Emerging research suggests that OEMs may lead to a reduction in the ovarian reserve, disrupt the hormonal environment, and impair folliculogenesis—each of which is essential for maintaining normal ovarian function and fertility [10]. For example, studies have linked OEMs to a decreased antral follicle count (AFC) and serum anti-Müllerian hormone (AMH) level, both of which serve as key markers of the ovarian reserve [11]. Furthermore, the surgical excision of OEMs may also compromise ovarian function by inadvertently reducing healthy ovarian tissue, thus raising concerns regarding long-term fertility implications [12,13].

**Figure 1:**
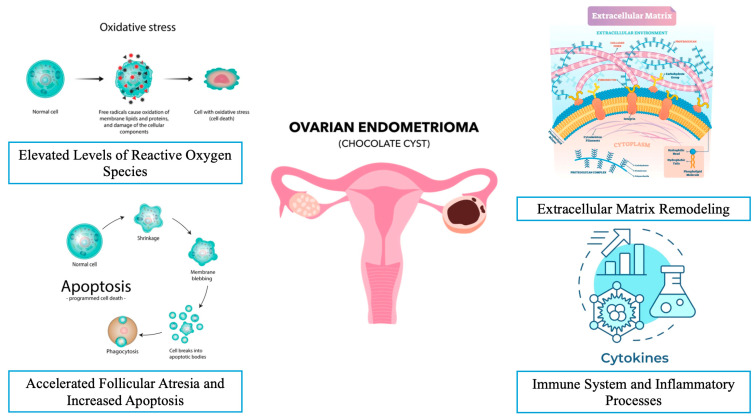
Possible mechanisms responsible for ovarian damage in the presence of endometrioma.

Understanding the physiological effects of OEMs on ovarian function is vital for optimizing fertility treatment strategies, particularly for women undergoing assisted reproductive technologies (ARTs). There is an ongoing debate around the impact of endometriosis on in vitro fertilization–embryo transfer treatment [14]. Several studies suggest that although the clinical pregnancy rates are lower, especially in those with advanced endometriosis, the live birth rates do not seem to be affected based on meta-analyses and data from national ART databases [15,16]. Furthermore, the presence of endometrioma itself does not change the clinical pregnancy and live birth rates [16]. Whether endometriosis impacts the oocyte and embryo quality is also controversial. The overall opinion is that the presence of endometriosis does reduce the oocyte and embryo quality, but this does not seem to translate into a clinical impact, as demonstrated by the similar live birth rates [17]. This section will delve deeper into how OEMs impact follicular development and the overall ovarian reserve (Table 1 and Figure 1).

### 3.1. The Effect of Endometrioma on the “Two-Cell-Two-Gonadotropin Theory” and Folliculogenesis

Ovarian follicles consist of several components, including an oocyte, granulosa cells (GCs), and theca cells (TCs), along with a follicular antrum that fills with follicular fluid (FF) as the follicle matures. For a high-quality, mature oocyte to develop, these biological structures must work in harmony. Any disruptions in this intricate balance can adversely affect a woman’s reproductive capacity [18,19].

Granulosa cells are essential players in the process of follicular development. They provide the necessary energy and materials for oocyte growth, regulate meiotic processes, and enhance the oocyte’s resistance to oxidative stress. However, the presence of OEMs can have adverse effects on GCs via induced apoptosis and autophagy, promotion of inflammation and fibrosis, and increased oxidative stress and decreased angiogenesis within the ovarian environment [20,21,22,23]. Additionally, the OEMs can disrupt steroid hormone synthesis and secretion, affecting the normal hormonal balance that is required for ovarian function [24,25,26,27,28,29]. These changes can impair the energy metabolism in cellular organelles like mitochondria and the endoplasmic reticulum, further complicating follicular health [26,30,31].

While theca cells are often studied less than GCs, they are crucial for hormone production. Theca cells primarily produce androgens in response to luteinizing hormone (LH) stimulation, which GCs convert into estrogens through the action of aromatase. TCs also produce growth-regulatory factors such as bone morphogenic proteins (BMPs) and transforming growth factor-β [32,33]. Recent studies have suggested a potential link between endometriosis and TCs. For instance, Casalechi et al. noted hormonal variations, such as differences in the adiponectin levels secreted by TCs, in endometriosis patients, which may impact follicular health and development [34]. BMPs, particularly BMP-15, play a vital role in ovarian follicle growth and differentiation. Notably, studies have shown that the BMP-15 levels in follicular fluid from patients with OEMs were significantly elevated compared to control groups, indicating a potential protective mechanism, possibly provided by the TCs, against the detrimental effects of OEMs [35,36].

### 3.2. Endometriomas and Ovarian Microenvironment

The ovarian reserve is defined as the functional capacity of the ovary, which is determined by both the quantity and quality of oocytes that are available for potential fertilization. It serves as a crucial indicator of a woman’s reproductive lifespan and her ability to conceive. Understanding how OEMs influence the ovarian reserve is therefore critical for both clinical practice and patient counseling.

A critical analysis in this article highlights the major pathophysiological mechanisms that negatively affect fertility in terms of oocyte quantity and quality and the subsequent embryo development in the presence of OEMs. All potential pathophysiological mechanisms are commonly related to the molecular milieu inside an OEM, the nature of the wall of the OEM, and the changes in the environment around the OEM.

#### 3.2.1. The Effect of Elevated Levels of Reactive Oxygen Species (ROS)

The deleterious effect of the presence of OEMs on the surrounding healthy tissue could be considered as one of the specific mechanisms. The concentrations of free iron in OEMs are reported to be higher because of the nature of the fluid in endometriotic cysts [37]. The source of iron is hemoglobin destruction via macrophages. Iron physiologically plays a key role in several cellular functions, including energy metabolism and oxygen transport. But a supraphysiological level of free iron could provide the production of ROS (superoxide anion, hydrogen peroxide, and hydroxyl radicals) by means of the Fenton reaction [38]. There is a balance between the ROS and cellular antioxidant defenses in the cell. This balance could be disturbed when the level of ROS exceeds the capacity of the cellular antioxidant. This is called oxidative stress (OS). The resultant OS could create a detrimental effect on the cellular function via the alteration of gene expression, growth and angiogenic factors, pro-inflammatory cytokines, signaling pathways, and adhesion molecules due to the unstable and highly reactive nature of ROS [39]. Additionally, significantly higher ROS production affects the molecular components of the cell via dysregulation of protein synthesis and membrane architecture, mitochondrial DNA damage, the depletion of ATP, dynamic instability of microtubules, inhibition of polymerization, enhancement of the depolymerization of microtubules, and, finally, chromosomal aberrations [40,41,42]. ROS can directly damage nuclear and mitochondrial DNA by inducing single- and double-strand breaks, as well as chemical modifications to DNA bases, such as the formation of 8-hydroxy-2′-deoxyguanosine (8-OHdG), a prominent marker of oxidative DNA damage. These alterations can lead to mutations, impaired DNA repair mechanisms, and genomic instability, further contributing to cellular dysfunction and disease pathogenesis. The interaction of ROS with DNA is particularly detrimental due to its potential to disrupt transcriptional fidelity and promote carcinogenic transformations [43]. In the context of female fertility, pathogenic variants in essential regulators of DNA damage repair, such as BRCA1, BRCA2, and other key genes involved in homologous recombination and non-homologous end-joining, play a critical role in maintaining genomic integrity during oogenesis. Dysregulation of these pathways can lead to meiotic errors, reduced oocyte quality, and early embryonic lethality, which are particularly relevant in conditions such as OMA (oocyte maturation arrest). Understanding the molecular mechanisms by which these variants impair DNA repair and their impact on fertility is crucial for developing targeted therapeutic strategies to improve reproductive outcomes in affected patients [44]. Moreover, the detrimental impact of ROS on the reproductive physiology, including ovarian steroidogenesis, oocyte maturation, ovulation processes, and blastocyst formation, should be taken into consideration because of the cytotoxic effects of ROS [45].

Endometriomas also influence the dynamics of FF. They tend to increase the OS and inflammation within the ovarian environment [21,46]. Elevated levels of iron and ferritin in endometrioma fluid and adjacent follicular fluids have been well documented. A systematic review by Wyatt et al. reported that all examined studies indicated increased levels of iron and iron-related proteins in endometriotic fluid compared to other types of ovarian cysts. They also noted localized iron overload in and around endometriotic lesions [47]. In instances where elevated FF iron levels are present, in vitro maturation rates of oocytes have shown a decline [48]. Increased ROS within the FF may contribute to this reduction in quality, although findings have been mixed across studies [49]. Singh et al. reported elevated ROS levels in FF from affected patients [50], while Nakagawa et al. found no significant differences in oxidative stress between FF in patients with unilateral endometriomas and those without [51]. An alternative perspective was provided by Regiani et al., who conducted a proteomic analysis using mass spectrometry on FF near endometriomas, suggesting that the protein profile in these patients promotes high OS levels [52]. Furthermore, iron overload and the resulting oxidative stress can trigger local inflammation. Numerous studies have documented elevated levels of cytokines such as IL-1 beta, IL-6, IL-8, and monocyte chemoattractant protein-1 in the follicular fluid from ovaries affected by endometriomas [53,54].

**Table 1:** Summary of the pathophysiology of endometrioma-mediated ovarian damage.

Pathophysiology	Effects on Ovarian Function and Microenvironment
Elevated levels of ROS [38,39,40,49,50]	Dysregulation of protein synthesis and membrane architecture Mitochondrial DNA damage Depletion of ATP Dynamic instability of microtubules Chromosomal aberrations Triggered local inflammation
Accelerated follicular atresia and increased apoptosis [51,52,53,54,55,56,57,58]	Decline in AMH levels Premature activation of primordial follicles
Activation of the plasminogen system and matrix metalloproteinases [59,60,61,62,63,64]	Degradation of ovarian cortex close to the endometrioma Increased proteolytic activity Excessive extracellular matrix remodeling leads to invasion and fibrosis
Chronic inflammation and increased levels of cytokines [65,66,67,68,69,70,71,72,73,74,75]	Promotion of angiogenesis Escape of ectopic endometrial tissue from the host immune system Proliferation of stromal cells

ROS: reactive oxygen species; DNA: deoxyribonucleic acid; ATP: adenosine triphosphate; AMH: anti-Müllerian hormone.

#### 3.2.2. Accelerated Follicular Atresia and Increased Apoptosis in the Presence of Endometrioma

It has been hypothesized that localized inflammation in the ovarian cortex associated with endometriomas can disrupt the delicate ovarian microenvironment by promoting aberrant follicle recruitment and accelerating follicular atresia, ultimately impairing antral follicle development. This inflammatory state may contribute to a significant reduction in the antral follicle count, which appears to correlate with a localized decline in AMH levels. AMH, a critical paracrine regulator, plays a pivotal role in maintaining the dormancy of primordial follicles. The diminished availability of AMH may lead to the premature activation of primordial follicles, rendering them vulnerable to atresia and further depleting the ovarian reserve [55,56].

In a landmark study conducted by Kitajima et al., the researchers aimed to investigate the effects of OEMs on early follicular development. The findings revealed a significantly higher percentage of atretic follicles in the cortex of ovaries with endometriomas compared to unaffected contralateral ovaries (20.3% vs. 6.3%), as confirmed by immunostaining. Additionally, the proportion of primordial follicles in the cortex of ovaries that were affected by endometriomas was considerably lower than in contralateral cyst-free ovaries (34% vs. 54%). When examining the developmental stage of early follicles, there was a notably higher rate of transitional follicle activation in cortex samples from endometriomas compared to contralateral cyst-free ovaries (82% vs. 63%). This study was the first to indicate that early follicular development might be prematurely activated, and that follicular atresia is more pronounced in ovaries with endometriomas compared to their contralateral counterparts, potentially resulting in a reduction in the ovarian reserve, a phenomenon referred to as the “burnout” hypothesis [57]. The localized depletion of primordial follicles in endometriomas is thought to result from focal inflammation, fibrosis, and the loss of cortex-specific stroma during disease progression. Recent studies have highlighted the activation of key signaling pathways, including phosphoinositide 3-kinase (PI3K)/protein kinase B (Akt)/mechanistic target of rapamycin (mTOR), Yes-associated protein (YAP), and transcriptional co-activator with PDZ-binding motif (TAZ), in human oocytes from ovaries that are affected by endometriomas. The PI3K/AKT/mTOR pathway plays a critical role in regulating cell survival, proliferation, and metabolism, and its dysregulation has been implicated in the pathogenesis of endometriosis. In endometriomas, the overactivation of this pathway may contribute to aberrant follicular activation and accelerated depletion of the ovarian reserve, providing mechanistic support for the “burnout” hypothesis [58,59]. Targeting this pathway could offer a potential therapeutic strategy to mitigate ovarian damage in endometriosis patients, as suggested by recent research on kinase signaling pathways in endometriosis [60].

In a cohort study conducted by Kasapoğlu et al., the researchers assessed the ovarian reserve in women with endometriomas over a six-month period. The results demonstrated a significant decline in AMH levels and AFC among endometrioma patients compared to control groups, highlighting the detrimental impact of endometriomas on the ovarian reserve [61]. A meta-analysis by Muzii et al. corroborated these findings, revealing significantly lower AMH levels in patients with endometriomas compared to those with benign ovarian cysts or those without endometriosis altogether [62]. Additionally, a recent comprehensive meta-analysis reported a notable decrease in AFC in endometrioma-affected ovaries when compared to healthy contralateral ovaries, further underscoring the negative implications of endometriomas on the ovarian reserve [63].

#### 3.2.3. Extracellular Matrix Remodeling and Fibrosis in the Presence of Endometrioma

The plasminogen activation system and matrix metalloproteinases (MMPs) play pivotal roles in the degradation and remodeling of the extracellular matrix (ECM). The plasminogen activation system consists of a network of proteolytic enzymes, with its central step being the extracellular conversion of the inactive plasminogen to plasmin, a broad-spectrum serine protease. The aberrant expression of components within this system has been implicated in pathological processes such as tissue invasion and fibrosis. Notably, plasminogen activator inhibitor-1 (PAI-1) regulates ECM degradation, promoting the accumulation of matrix structural elements that drive fibrotic responses. These responses are characterized by the recruitment of inflammatory cells, macrophages, and myofibroblasts [64]. In patients with endometriosis, the PAI-1 mRNA expression is significantly elevated in both the eutopic endometrium and endometriotic lesions compared to the normal endometrium [65]. Supporting this, Boss et al. demonstrated significantly higher concentrations of PAI-1 in endometrioma fluid compared to benign ovarian cysts (247 vs. 37 μg/L). Additionally, the levels of urokinase plasminogen activator (uPA) and PAI-2 were markedly elevated in endometrioma fluid compared to cyst fluid from malignant ovarian tumors (155 vs. 21.7 μg/L and 199 vs. 12.2 μg/L, respectively) [66]. The high concentrations of these substances in endometrioma fluid contribute to the degradation of surrounding tissues, exacerbating disease progression and fibrosis.

Matrix metalloproteinases are zinc-dependent endopeptidases and are essential for ECM remodeling, with their activity being tightly regulated by tissue inhibitors of metalloproteinases (TIMPs). Increased expressions of MMP-1 and MMP-9 have been observed in ovarian endometriotic tissue [67,68]. Luddi et al. analyzed the molecular profiles of MMP-2, MMP-3, and MMP-10, along with their inhibitors TIMP-1 and TIMP-2, in the healthy endometrium and eutopic endometrium from women with endometriomas. Their study revealed significant upregulation of all investigated MMPs and TIMPs in OEMs [69]. These alterations resulted in enhanced proteolytic activity and excessive ECM remodeling, contributing to the invasive and fibrotic characteristics of endometriomas.

It is very well known that ECM remodeling generated via activated platelets, macrophages, and myofibroblasts contributes to the formation of fibrosis during the repair of inflamed or damaged tissue. The increase in the level of transforming growth factor (TGF-β) and the deposition of collagen are primarily responsible for the biological process of fibrosis. OEMs are surrounded by fibrotic tissue known as a pseudo-capsule because of OEM-associated fibrogenesis. Activated platelets that produce important growth factors and cytokines such as TGF-β, platelet-derived growth factor (PDGF), and epidermal growth factor (EGF) also play a key role in the onset of tissue fibrosis related to OEM [70].

#### 3.2.4. The Immune System and Inflammatory Processes in the Presence of Endometrioma

Endometriosis is recognized as a chronic, inflammatory condition characterized by both local and systemic inflammatory processes, which play a critical role in the pathogenesis of endometriomas. The role of the immune system and inflammatory processes is also critical in the pathogenesis of endometriosis. These inflammatory mediators not only promote angiogenesis but also support the survival of ectopic endometrial tissue, further complicating the condition. Key contributors to the inflammatory milieu in endometriomas include alterations in serum and local cytokine levels, particularly interleukin-6 (IL-6), interleukin-8 (IL-8), and interleukin-1β (IL-1β), alongside dysfunctions in the innate immune system. Among the numerous signaling pathways that have been proposed to be involved in these processes, the NF-κB pathway has been conclusively demonstrated to be a central player in mediating the inflammatory responses linked to endometriomas [71].

Elevated levels of IL-6 have been consistently reported in both the blood serum and tissues of endometriomas and their surrounding environment [21,72,73]. IL-6 exerts a range of immunomodulatory effects that contribute to the chronic inflammatory state. Firstly, it impairs the cytotoxic function of natural killer (NK) cells, thereby weakening immune surveillance [74]. Additionally, IL-6 facilitates the transition from acute to chronic inflammation by promoting a shift from a neutrophil-dominated infiltrate, typical of acute inflammation, to one dominated by monocytes and macrophages, characteristic of chronic inflammation [75]. Furthermore, elevated serum IL-6 levels have been associated with unexplained infertility [76]. This may be due to its inhibitory effects on blastocyst formation, as observed in animal models [77].

Like IL-6, IL-8 levels are significantly elevated in both the serum and tissues of patients with endometriomas [78]. As a pro-inflammatory cytokine, IL-8 plays a crucial role in maintaining the inflammatory microenvironment. Beyond its inflammatory effects, IL-8 also promotes the proliferation of stromal cells, contributing to the pathological remodeling and progression of endometriotic lesions [79]. Additionally, interleukin-1β (IL-1β), another key pro-inflammatory cytokine, has been found at elevated levels in endometrioma tissues [21]. IL-1β is known to stimulate the production of other inflammatory mediators, such as IL-6 and tumor necrosis factor-alpha (TNF-α), which further exacerbate the proliferation of endometrial stromal cells and tissue inflammation [80]. Moreover, IL-1β and vascular endothelial growth factor (VEGF) reduce apoptosis and decrease Bax expression in endometrial epithelial cells from patients with endometriosis [81]. This cytokine also activates the NF-κB pathway [82], amplifying the inflammatory cascade within endometriomas and promoting the chronic inflammatory state that is characteristic of the disease. These cytokine-driven processes underscore the intricate interplay between inflammation and immune dysfunction in the pathophysiology of endometriomas, highlighting potential therapeutic targets for managing this condition.

## 4. The Impact of Endometriomas on Assisted Reproductive Technology Outcomes

The impact of endometriomas on the ovarian response to exogenous gonadotropins, embryo quality, and reproductive outcomes during IVF treatment remains a topic of ongoing debate in the field, as previously noted [83]. Results from various studies present a mixed picture: some report higher rates of unexpected poor responses, lower numbers of oocytes retrieved, and lower live birth rates in patients with endometrioma [84], while others find no significant difference in terms of pregnancy rates [85,86], and a few even report higher fertilization rates among those with endometriomas [87]. Recently, Lafuente et al. published a systematic review and meta-analysis that evaluated indirect markers of oocyte quality in patients with OEMs undergoing IVF or in vitro cytoplasmic sperm injection (ICSI). Their findings indicated no significant difference in fertilization or blastulation rates between patients with and without OMA. This comprehensive study was noteworthy for being the first meta-analysis to examine blastulation rates in this specific context [88]. While the latest meta-analysis suggests that endometriomas do not significantly affect oocyte quality based solely on indirect markers, earlier studies have indicated considerable changes in the morphological structure and transcriptomic profile of oocytes from patients with endometriomas [89,90,91].

In conclusion, endometriomas can negatively impact the ovarian reserve, oocyte and embryo quality, and success of oocyte retrieval during IVF treatment. Nevertheless, despite these adverse effects, endometriomas do not appear to significantly affect the live birth rate following IVF (Table 2). However, for individuals attempting to conceive naturally, the detrimental effects of endometriomas on oocyte and embryo quality may present challenges in achieving pregnancy.

**Table 2:** Myths and facts regarding endometrioma-mediated ovarian damage.

Myths	Facts
Myth: Endometriomas do not significantly affect the ovarian reserve.	Fact: Endometriomas are associated with a reduction in the ovarian reserve, evidenced by decreased AFC and AMH levels [15,16,61,62,63].
Myth: Endometriomas do not affect the oocyte or embryo quality.	Fact: Endometriomas are linked to reduced oocyte and embryo quality due to oxidative stress, inflammation, and altered follicular fluid composition [21,43,50,89,90,91].
Myth: Endometriomas do not influence IVF outcomes.	Fact: While live birth rates are not significantly affected, endometriomas are associated with a lower number of retrieved oocytes and poor ovarian response during IVF [84,85].
Myth: Oxidative stress in endometriomas has no significant impact on ovarian function.	Fact: Elevated ROS in endometriomas cause mitochondrial DNA damage, ATP depletion, chromosomal aberrations, and impaired folliculogenesis [40,41,42,43,45].
Myth: Endometriomas do not accelerate follicular atresia.	Fact: Endometriomas promote the premature activation of primordial follicles and accelerate follicular atresia, leading to a decline in the ovarian reserve [55,56,57].
Myth: Endometriomas do not alter the ovarian microenvironment.	Fact: Endometriomas induce chronic inflammation, fibrosis, and extracellular matrix remodeling, disrupting the ovarian microenvironment [65,66,67,68,69,70,71,72,73,74,75,76,77,78,79,80,81,82].
Myth: Endometriomas do not affect granulosa cell function.	Fact: Endometriomas impair granulosa cell steroidogenesis, induce apoptosis, and disrupt energy metabolism, negatively impacting follicular development [19,20,21,22,25,30,31].
Myth: Endometriomas do not influence theca cell function.	Fact: Endometriomas alter theca cell hormone production and the secretion of growth factors, such as BMP-15, which may impact follicular health [32,33,34,35,36].
Myth: Endometriomas do not trigger immune dysregulation.	Fact: Endometriomas are associated with elevated levels of pro-inflammatory cytokines (e.g., IL-6, IL-8, IL-1β) and immune dysfunction, contributing to disease progression [71,72,73,74,75,76,77,78,79,80,81,82].
Myth: Endometriomas do not cause fibrosis in the ovarian tissue.	Fact: Endometriomas are surrounded by fibrotic tissue due to excessive extracellular matrix remodeling and the activation of fibrogenic pathways (e.g., TGF-β, PDGF) [65,66,67,68,69,70].
Myth: Endometriomas do not affect the follicular fluid composition.	Fact: Endometriomas alter follicular fluid by increasing iron levels, ROS, and pro-inflammatory cytokines, which negatively impacts oocyte maturation and quality [21,37,45,46,47,48,49,50,51,52,53,54].
Myth: Endometriomas do not activate signaling pathways that harm ovarian function.	Fact: Endometriomas activate pathways such as PI3K/AKT/mTOR, YAP, and TAZ, leading to aberrant follicular activation and ovarian damage [56,57,58,59,60].

AFC: antral follicle count; AMH: anti-Müllerian hormone; IVF: in vitro fertilization; ROS: reactive oxygen species; DNA: deoxyribonucleic acid; ATP: adenosine triphosphate; BMP-15: bone morphogenic protein-15; IL-: interleukin; TGF-β: transforming growth factor beta; PDGF: platelet-derived growth factor; YAP: yes-associated protein; TAZ: transcriptional co-activator with PDZ-binding motif.

## 5. Conclusions

In summary, the presence of endometrioma seems to cause ovarian damage via several potential factors that induce oxidative stress, apoptosis, inflammation, and fibrosis; the disruption of folliculogenesis; and decreased angiogenesis within the ovarian microenvironment. However, endometriosis does not affect the chances of pregnancy during IVF treatment, despite an unexpectedly poor response and a retrieval of a lower number of oocytes. Although existing studies substantiate the premise that endometriomas induce ovarian damage, further research is essential to explore interventions that may minimize these adverse effects on ovarian function. Finally, while this review discusses key mechanisms such as oxidative stress, inflammation, and fibrosis, it does not comprehensively explore emerging areas such as epigenetic modifications, microbiome changes, or other molecular pathways that may contribute to endometrioma-mediated ovarian damage. Addressing these limitations in future research will be essential to deepen our understanding of the pathophysiology of endometrioma-mediated ovarian damage and improve clinical management strategies.

## Data Availability

No new data were created or analyzed in this study. Data sharing is not applicable to this article.
